# Fatal *Nocardia farcinica* Bacteremia Diagnosed by Matrix-Assisted Laser Desorption-Ionization Time of Flight Mass Spectrometry in a Patient with Myelodysplastic Syndrome Treated with Corticosteroids

**DOI:** 10.1155/2013/368637

**Published:** 2013-04-16

**Authors:** Christian Leli, Amedeo Moretti, Francesco Guercini, Angela Cardaccia, Leone Furbetta, Giancarlo Agnelli, Francesco Bistoni, Antonella Mencacci

**Affiliations:** ^1^Microbiology Section, Department of Experimental Medicine and Biochemical Sciences, Santa Maria della Misericordia Hospital, Sant'Andrea delle Fratte, 06132 Perugia, Italy; ^2^Stroke Unit and Division of Cardiovascular Medicine, Santa Maria della Misericordia Hospital, University of Perugia, 06132 Perugia, Italy

## Abstract

*Nocardia farcinica* is a Gram-positive weakly acid-fast filamentous saprophytic bacterium, an uncommon cause of human infections, acquired usually through the respiratory tract, often life-threatening, and associated with different clinical presentations. Predisposing conditions for *N. farcinica* infections include hematologic malignancies, treatment with corticosteroids, and any other condition of immunosuppression. Clinical and microbiological diagnoses of *N. farcinica* infections are troublesome, and the isolation and identification of the etiologic agent are difficult and time-consuming processes. We describe a case of fatal disseminated infection in a patient with myelodysplastic syndrome, treated with corticosteroids, in which *N. farcinica* has been isolated from blood culture and identified by Matrix-Assisted Laser Desorption-Ionization Time of Flight Mass Spectrometry. The patient died after 18 days of hospitalization in spite of triple antimicrobial therapy. *Nocardia farcinica* infection should be suspected in patients with history of malignancy, under corticosteroid therapy, suffering from subacute pulmonary infection,and who do not respond to conventional antimicrobial therapy. Matrix-Assisted Laser Desorption-Ionization Time of Flight Mass Spectrometry can be a valuable tool for rapid diagnosis of nocardiosis.

## 1. Introduction

*Nocardia *infections are rare but potentially fatal, typically occurring in patients with cell-mediated immunosuppressive conditions, but occasionally in immunocompetent patients as well [1].

*Nocardia farcinica *is a Gram-positive branching filamentous bacillus causing localized and disseminated infections in humans, including pulmonary infections [2–9], subcutaneous [10] and brain abscesses [11–13], and bacteremia [14–19], especially in immunocompromised patients [20].

The microbiological diagnosis of nocardiosis and the identification of *Nocardia* clinical isolates by conventional methods are difficult and time-consuming processes [21]. Polymerase chain reaction has been employed for *Nocardia* spp. identification from different clinical specimens [22, 23]. Recently, Matrix-Assisted Laser Desorption-Ionization Time of Flight Mass Spectrometry (MALDI-TOF MS) has been developed for rapid identification of the majority of human pathogens, but hitherto it has been rarely used for *Nocardia* identification [24, 25].

We describe a lethal case of *N. farcinica* disseminated infection in a patient with myelodysplastic syndrome treated with corticosteroids, in which the etiologic agent was isolated from blood culture and rapidly identified by MALDI-TOF MS.

## 2. Case Presentation

In July 2012, a 91-year-old Italian male patient was admitted to the Internal Medicine Unit of the Perugia General Hospital complaining from fatigue, anorexia, weight loss of 10 Kg in few weeks, and arthralgia. The patient's medical history revealed that he was suffering from myelodysplastic syndrome, complicated by autoimmune hemolytic anemia since March 2012, treated with periodic blood transfusions, alpha recombinant human erythropoietin 40,000 IU/week subcutaneously, and since the last month, with prednisone 25 mg/day, cutting down the dosage by half every week. The patient completed corticosteroid therapy few days before admission. He suffered also from previous ischemic stroke, moderate renal insufficiency, psychosis, and chronic obstructive pulmonary disease.

On admission the examination showed low-grade fever (37.2°C), fatigue, nonproductive cough, bilateral basal crackles, and wheezes at chest auscultation. His blood pressure was 118/78 mmHg, the heart rate was 82 beats per minute, the respiratory rate was 20 breaths per minute, and oxygen saturation measured by pulse oximetry was 95%. The patient's body weight was 76 kg. No other significant abnormalities were found. Two sets of blood cultures were drawn from different peripheral sites at the same time.

**Figure 1:**
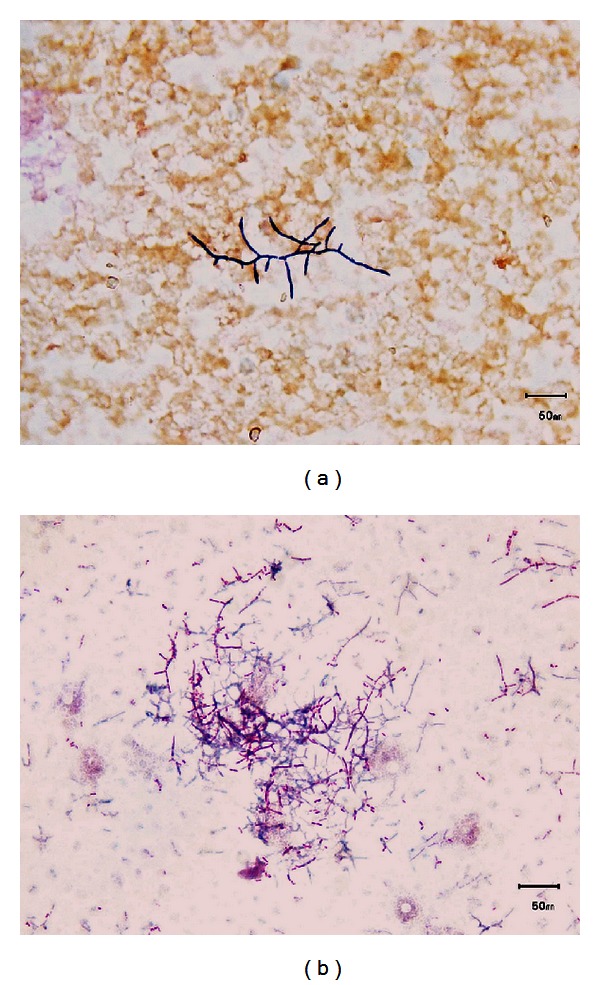
(a) Gram stain of blood culture showing a cluster of beaded branching filamentous Gram-positive rods. (b) Modified acid fast Kinyoun staining of blood culture showing filamentous red-stained partially acid-fast rods.

**Figure 2:**
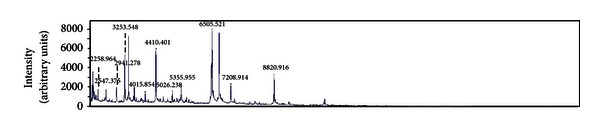
Mass spectral profile of the *Nocardia farcinica* isolate obtained from colonies grown on Columbia blood agar after 48 hrs of incubation.

Main laboratory tests performed on admission showed hemoglobin 10.2 g/dL, leukocytes 11.3 × 10^3^/*μ*L (neutrophils 77%, lymphocytes 16%, and monocytes 6.3%), platelets 202 × 10^3^/*μ*L, sedimentation rate 42 mm/h, creatinine 1.95 mg/dL, albumin 2.7 g/dL, and gamma globulin 29.5% with two monoclonal bands in gamma region. Other blood-chemistry tests did not show significant pathological values. The chest X-ray performed on admission disclosed multiple nodular acinar opacities throughout both lungs with diffuse interstitial thickening. The Mantoux skin test showed negative result. A sputum sample was collected, and Gram stain showed more than 25 neutrophils per low-power field and normal upper respiratory tract bacterial population, but no potential pathogens present. Culture was negative for common bacterial pathogens. A presumptive diagnosis of community-acquired pneumonia in immunocompromised patient was made, and antimicrobial therapy with ceftriaxone 2 g intravenously/die was started. In spite of that, the patient's conditions slightly worsened over the next couple of days, with decreased level of consciousness and low-grade fever (≤37.5°C) episodes every day. On the fourth day, the aerobic bottle of the first blood culture set flagged positive. Gram staining of positive bottle revealed branching Gram-positive rods (Figure 1(a)) that were also weakly acid-fast on modified Kinyoun stain (Figure 1(b)). On the basis of these findings, *Nocardia *spp. bacteremia was suspected and ceftriaxone was replaced with trimethoprim/sulfamethoxazole 160/800 mg intravenously three times daily, plus linezolid 600 mg intravenously twice daily. Positive blood culture was subcultured, and after 2 days of incubation very small colonies appeared on Columbia blood agar and were identified by MALDI-TOF MS (Bruker Daltonics, Bremen, Germany) as *Nocardia farcinica*, with a score of 1.879 (Figure 2). On the fifth day after subculturing, *N. farcinica* identification was confirmed by conventional biochemical tests. The isolate was susceptible to trimethoprim/sulfamethoxazole, linezolid, ciprofloxacin, and amikacin but resistant to ceftriaxone, clarithromycin, and imipenem. Ciprofloxacin 200 mg intravenously twice daily was added. Sputum, collected again soon after presumptive diagnosis of nocardiosis, was cultured for *Nocardia* spp., but resulted negative after 10 days of incubation.

**Figure 3:**
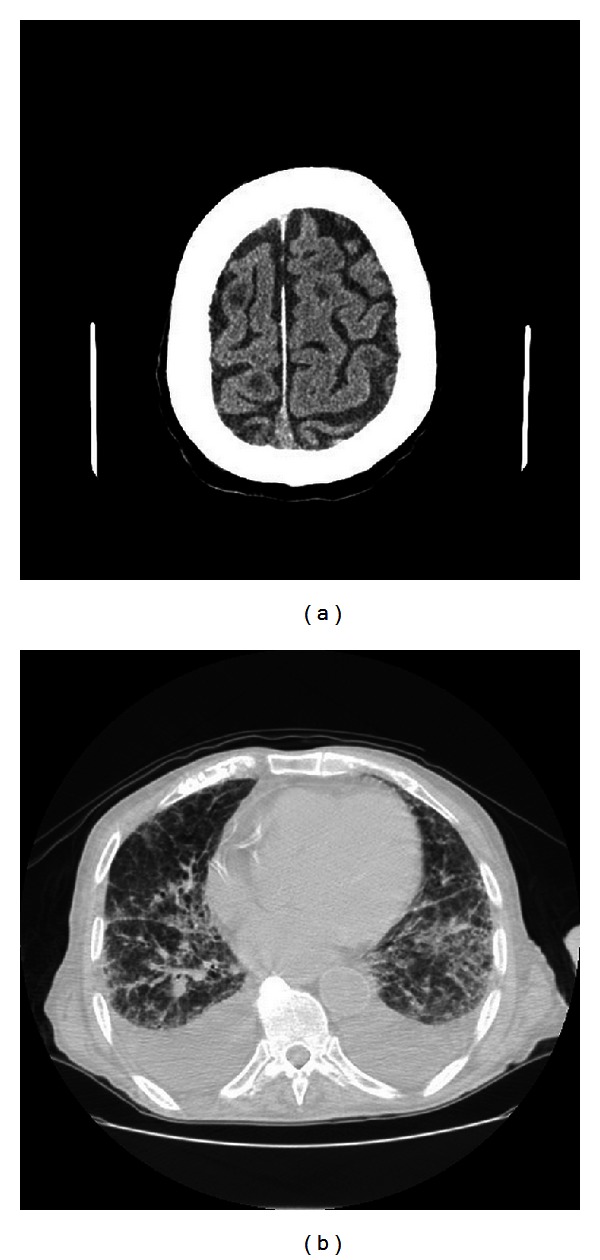
(a) Computerized tomography (CT) scan of the patient's brain showing multiple enhancing focal lesions. (b) Chest CT scan showing multiple nodular lesions and bilateral pleural effusion.

On day 10 the patient's level of consciousness deteriorated to a state of light coma, difficult to awaken, with worsening shortness of breath. Oxygen saturation measured by pulse oximetry showed values of 89%; therefore oxygen therapy was started via nasal cannula at 2 liters per minute (FiO_2_ 28%). A computerized tomography (CT) scan of the brain was performed, and it showed multiple enhancing focal lesions in the frontal regions (Figure 3(a)), midbrain, and in the cerebellum, and a chest CT scan showed multiple nodular lesions and bilateral pleural effusion (Figure 3(b)). Unfortunately, the patient was suffering from psychosis, and although in a coma, he was uncooperative when awakened; therefore a thoracentesis could not be performed. For the same reason, the patient refused noninvasive mechanical ventilation, and oxygen therapy was continued via nasal cannula and then via Venturi mask with further increases. The patient died on day 18 of hospitalization.

## 3. Discussion

*Nocardia farcinica *is a Gram-positive actinomycete saprophyte, ubiquitous in the environment. It is considered an opportunistic pathogen that affects patients with impaired cell-mediated immunity. Most of the patients with *N. farcinica *infection had predisposing factors like hematologic malignancies, treatment with corticosteroids, solid tumors, bone marrow or solid organ transplantation, HIV infection, chronic pulmonary, and renal diseases [16]. The most common clinical presentation is a subacute pneumonia with nodules, necrosis, cavitation, or lung abscesses [2–5]. Due to nonspecific clinical and radiologic manifestations, pulmonary nocardiosis is easily misdiagnosed as tuberculosis or bacterial pneumonia [26]. Hematogenous dissemination to central nervous system in the form of brain abscesses [11–13] or to other sites can occur [10]*. Nocardia farcinica *bacteremia has been rarely described [14, 18, 27]. It is associated with a very poor prognosis [14] and poses a significant clinical and diagnostic challenge [18].

Also in this case, the clinical presentation on admission was aspecific, leading to the initial diagnosis of bacterial pneumonia in an immunocompromised patient without suspicion of nocardiosis. In our patient, the diagnosis, suspected on the Gram and Kinyoun staining of the positive blood culture, was definitively confirmed two days later by MALDI-TOF MS. Identification by conventional standard methods was obtained only five days after blood culture positivization. To the best of our knowledge, MALDI-TOF MS was recently employed to identify different *Nocardia* spp. from bacterial collections [24, 25], but not from human clinical specimen. This is the first report in which this method has been used during the routine laboratory practice to identify a *Nocardia *clinical isolate, recovered from blood culture, shortening the diagnosis of disseminated nocardiosis. Moreover, since *N. farcinica* exhibits a high degree of resistance to many antimicrobials, the early identification obtained by MALDI-TOF MS was of high importance.

Nevertheless, we were unable to recover the microorganism from patient's sputum, confirming that *Nocardia* spp. are difficult to isolate from this kind of sample even in documented cases of pulmonary infection, because of the low number of bacteria in the sample and/or the slow growth and the overgrowth by the contaminating bacteria [28].

The isolate was susceptible only to ciprofloxacin, linezolid, and trimethoprim/sulfamethoxazole, showing a typical pattern of antimicrobial resistance. *Nocardia farcinica* is often susceptible to amoxicillin/clavulanic acid, amikacin, imipenem, and ciprofloxacin, and differently from other species, resistant to ampicillin, cephalosporins, erythromycin, gentamicin, and tobramycin. The antimicrobial resistance, together with the tendency to disseminate, is considered responsible for the high mortality of *N. farcinica* infections [16]. These intrinsic characteristics of the organism, together with several comorbidities (myelodysplastic syndrome, renal insufficiency, and chronic obstructive pulmonary disease), long-term prednisone theraphy, and the late etiological diagnosis of the infection, explain the unfavorable outcome for our patient.

## 4. Conclusion

This case shows that *N. farcinica* infection should be suspected in patients with history of malignancy, under corticosteroid therapy, suffering from subacute pulmonary infection, that does not respond to conventional antimicrobial therapy. Matrix-Assisted Laser Desorption-Ionization Time of Flight Mass Spectrometry is a valuable tool for a more rapid microbiological diagnosis of nocardiosis.

## References

[1] Wilson JW (2012). Nocardiosis: updates and clinical overview. *Mayo Clinic Proceedings*.

[2] Bruno P, Ricci A, Pezzuto A, Martone L, Gencarelli G, Mariotta S (2011). Severe pneumonia caused by *Nocardia farcinica* and complicated by *Staphylococcus haemoliticus* superinfection. *European Review for Medical and Pharmacological Sciences*.

[3] Babayigit A, Olmez D, Sozmen SC (2010). Infection caused by *Nocardia farcinica* mimicking pulmonary metastasis in an adolescent girl. *Pediatric Emergency Care*.

[4] De La Iglesia P, Viejo G, Gomez B, De Miguel D, Del Valle A, Otero L (2002). Fatal pulmonary *Nocardia farcinica* infection. *Journal of Clinical Microbiology*.

[5] Mari B, Montón C, Mariscal D, Luján M, Sala M, Domingo C (2001). Pulmonary nocardiosis: clinical experience in ten cases. *Respiration*.

[6] Beucher J, Belleguic C, Brinchault G, Deneuville E, Donnio PY, Roussey M (2010). *Nocardia farcinica* infection in a patient with cystic fibrosis. *Revue des Maladies Respiratoires*.

[7] Bittar F, Stremler N, Audié JP (2010). *Nocardia farcinica* lung infection in a patient with cystic fibrosis: a case report. *Journal of Medical Case Reports*.

[8] Petersen BE, Jenkins SG, Yuan S, Lamm C, Szporn AH (2007). *Nocardia farcinica* isolated from bronchoalveolar lavage fluid of a child with cystic fibrosis. *The Pediatric Infectious Disease Journal*.

[9] Brasileiro RMF, Pinho ACCDA, Medeiros CS (2007). Pulmonary nocardiosis in a patient who was a chronic corticosteroid user. *Revista da Sociedade Brasileira de Medicina Tropical*.

[10] Yokota S, Kawabe K, Yamada H, Nunomura M (2010). A case of subcutaneous abscess caused by *Nocardia farcinica* in an aplastic anemia patient. *Nihon Ishinkin Gakkai Zasshi*.

[11] Scharfen J, Morávková M, Bunček M (2010). *Nocardia farcinica* as the causative agent of a brain abscess in a patient with interstitial lung disease. *Epidemiologie, Mikrobiologie, Imunologie*.

[12] Iannotti CA, Hall GS, Procop GW, Tuohy MJ, Staugaitis SM, Weil RJ (2009). Solitary *Nocardia farcinica* brain abscess in an immunocompetent adult mimicking metastatic brain tumor: rapid diagnosis by pyrosequencing and successful treatment. *Surgical Neurology*.

[13] Fihman V, Berçot B, Mateo J (2006). First successful treatment of *Nocardia farcinica* brain abscess with moxifloxacin. *The Journal of Infection*.

[14] Christidou A, Maraki S, Scoulica E, Mantadakis E, Agelaki S, Samonis G (2004). Fatal *Nocardia farcinica* bacteremia in a patient with lung cancer. *Diagnostic Microbiology and Infectious Disease*.

[15] Al-Tawfiq JA, Al-Khatti AA (2010). Disseminated systemic *Nocardia farcinica* infection complicating alefacept and infliximab therapy in a patient with severe psoriasis. *International Journal of Infectious Diseases*.

[16] Torres OH, Domingo P, Pericas R, Boiron P, Montiel JA, Vázquez G (2000). Infection caused by *Nocardia farcinica*: case report and review. *European Journal of Clinical Microbiology & Infectious Diseases*.

[17] Bhave AA, Thirunavukkarasu K, Gottlieb DJ, Bradstock K (1999). Disseminated nocardiosis in a bone marrow transplant recipient with chronic GVHD. *Bone Marrow Transplantation*.

[18] Peters BR, Saubolle MA, Costantino JM (1996). Disseminated and cerebral infection due to *Nocardia farcinica*: diagnosis by blood culture and cure with antibiotics alone. *Clinical Infectious Diseases*.

[19] Farina C, Boiron P, Goglio A, Provost F (1995). Human nocardiosis in northern Italy from 1982 to 1992. Northern Italy Collaborative Group on Nocardiosis. *Scandinavian Journal of Infectious Diseases*.

[20] Brown-Elliott BA, Brown JM, Conville PS, Wallace RJ (2006). Clinical and laboratory features of the *Nocardia* spp. based on current molecular taxonomy. *Clinical Microbiology Reviews*.

[21] Versalovic J, Carroll K, Funke G (2011). *Manual of Clinical Microbiology*.

[22] Park BS, Park YJ, Kim YJ (2008). A case of disseminated *Nocardia farcinica* diagnosed through DNA sequencing in a kidney transplantation patient. *Clinical Nephrology*.

[23] Hasegawa T, Gonoi T, Ito J, Kogure T, Yazawa K, Mikami Y (2007). Identification of *Nocardia farcinica* by a PCR primer amplifying a specific DNA band for the bacterium. *Nihon Ishinkin Gakkai Zasshi*.

[24] Verroken A, Janssens M, Berhin C (2010). Evaluation of matrix-assisted laser desorption ionization-time of flight mass spectrometry for identification of *Nocardia* species. *Journal of Clinical Microbiology*.

[25] Farfour E, Leto J, Barritault M (2012). Evaluation of the andromas matrix-assisted laser desorption ionization-time of flight mass spectrometry system for identification of aerobically growing gram-positive bacilli. *Journal of Clinical Microbiology*.

[26] Agterof MJ, van der Bruggen T, Tersmette M, ter Borg EJ, van den Bosch JMM, Biesma DH (2007). Nocardiosis: a case series and a mini review of clinical and microbiological features. *The Netherlands Journal of Medicine*.

[27] Lai CC, Lee LN, Teng LJ, Wu MS, Tsai JC, Hsueh PR (2005). Disseminated *Nocardia farcinica* infection in a uraemia patient with idiopathic thrombocytopenia purpura receiving steroid therapy. *Journal of Medical Microbiology*.

[28] Palmer DL, Harvey RL, Wheeler JK (1974). Diagnostic and therapeutic considerations in *Nocardia asteroides* infection. *Medicine*.

